# Supplementary material for: Vitamin K1 and K2 in the Diet of Patients in the Long Term after Kidney Transplantation
Source: Nutrients. 2022 Nov 29;14(23):5070. doi: 10.3390/nu14235070 (PMC9741316; doi:10.3390/nu14235070)
Supplement: Supplementary file 1 [file nutrients-14-05070-s001.zip › nutrients-2041973-supplementary File S1.pdf]

Evaluation of vitamins K1 and K2 intake: in the diet and from supplements in kidney transplant recipients

The purpose of this questionnaire is to provide information about the amounts of vitamin K intake both in the diet as well as derived from supplements. The research will be extended to information about intake of anticoagulants and drugs that lower blood cholesterol and/or glycemia.

The questionnaire consists of two parts:

1. Patients data: sex, age, weight, height, earlier mentioned drugs and supplements intake.
   Respondent provides information about sex, weight, height, intake of anticoagulants or drugs lowering glycemia or cholesterol, and supplements. Respondent provides also information about medical history, including fractures and time of their appearance.
   Investigator takes weight measurements of the respondent using body composition analyzer TANITA BC – 545N and interprets the readout of respondent body mass index (BMI) from the analyzer.
2. Food intake diary: information about solid and liquid products consumed over three consecutive days, however excluding Saturdays and Sundays in order to allow for more accurate results, since during weekends respondents diet may differ from that consumed on working days.
   Food diary consists of two parts:
   A) Meals menu from three consecutive days presented by respondent
   B) Detailed products list that enables the respondent to best specify food products consumed which translates into more accurate results.

On the basis of the information gathered from the questionnaire a summary of vitamin K intake from supplements, solid and liquid products will be made. To estimate the exact vitamin K amount we will use the tables of vitamin content in food products from the National Food and Nutrition Institute complemented by American (www.nal.usda.gov) and Dutch (www.frida.fooddata.dk) data bases.
For research purposes we use a program recommended by the National Food and Nutrition Institute – Diet 5D.
Data derived from the respondents will also provide information about drug intake of particular anticoagulants and drugs lowering blood cholesterol and glycemia.
Gathered data will be used only for research purposes. The questionnaire is anonymous and voluntary. After completing the questionnaire, the investigator gives the respondent a data disclosure statement to sign.

Questionnaire – part I

Sex<br>□ Women<br>□ Men	Age………………………<br><br>Height……………………..<br><br>Weight…………………	BMI<br><br>…………………………………

1. When did you have kidney transplantation? ***(state year and month)***

…………………………………………………….

2. Have you ever had myocardial infarction?
□ no
□ yes
If the answer is yes, please state the date *(year, month)* ……………………………………...

3. Have you ever had brain stroke?
□ no
□ yes
If the answer is yes, please state the date *(year, month)* ……………………………………...

4. Have you ever had any fractures?
□ no
□ yes
If the answer is yes:
a) did the fracture occur before or after kidney transplanation?
□ before
□ after
b) which bone(s) was involved?
………………………………………………………………………………………
c) when did the fracture occur? *(year, month)*
…………………………………………………………………….

5. Are you taking drugs lowering blood cholesterol?
□ no
□ yes
If the answer is yes, what is the name of the drug?……………………………………………
At what daily dose do you take it?……………………………………………………………
Since when have you been taking it? *(year, month)* …………………………..………........
6. Are you taking drugs lowering glycemia?
□ no
□ yes
If the answer is yes, what is the name of the drug?……………………………………………
At what daily dose do you take it?……………………………………………………………
Since when have you been taking it? *(year, month)* …………………………..………........

7. Are you taking any anticoagulant drugs listed below?
□ Acenocumarol
□ Warfin
If the answer is yes, since when have you been taking it? *(year, month)*
…………………………..………........

8. Do you take any diet supplements?
□ no
□ yes
If the answer is yes, what is the name of the supplement?……………………………………………
At what daily dose do you take it?……………………………………………………………
Since when have you been taking it? *(year, month)* …………………………..………........

<u>Questionnaire– part II</u>

CONSUMED FOOD DIARY of the respondent on day one

Type of meal	Meal/product	Components	Measure	Mass (filled in by respondent)
**Breakfast**	Scrambled eggs with spinach and mushrooms, slice of bread with butter, green tea	Chicken eggs<br>Extra butter<br>Fresh spinach<br>Mushroom<br>Tomato<br>Rye bread<br>Green tea<br>White sugar	2 pieces<br>1 spoon<br>1 handful<br>4 pieces<br>1 piece<br>2 slices<br>1 cup<br>1 spoon	120<br>10<br>30<br>80<br>100<br>80<br>50<br>10
**Second breakfast**	An apple	Apple	1 piece	120
**Dinner**	Boiled potatos, roasted chicken, carrot salad, orange juice	Potatos<br>Chicken fillet<br>Olive oil<br>Carrot<br>Orange jucie	2 pieces<br>1 piece<br>1 spoon<br>1 piece<br>1 cup	150<br>120<br>10<br>45<br>250
**Teatime**	Tomato juice	Tomato juice	1 cup	250
**Supper**	Sandwich with butter, ham, tomato, black tea	Rye bread<br>Extra butter<br>Sopot's sirloin<br>Tomato<br>Black tea<br>White sugar	2 slices<br>1 spoon<br>2 slices<br>4 slices<br>1 cup<br>2 teaspoon	80<br>10<br>20<br>50<br>250<br>10
**Additional meals and liquids, herbs including**	Kefir	Kefir	1 cup	250

CONSUMED FOOD DIARY of the respondent on day two

Type of meal	Meal/product	Components	Measure	Mass (filled in by respondent)
**Breakfast**				
**Second breakfast**				
**Dinner**				
**Teatime**				
**Supper**				
**Additional meals and liquids, herbs including**				

CONSUMED FOOD DIARY of the respondent on day three

Type of meal	Meal/product	Components	Measure	Mass (filled in by respondent)
**Breakfast**				
**Second breakfast**				
**Dinner**				
**Teatime**				
**Supper**				
**Additional meals and liquids, herbs including**				

PRODUCTS – DETAILED LIST

**CEREALS**	**Bread**	**Dairy + % fat**
□ Oats<br>□ Millet<br>□ Buckwheat<br>□ Corn<br>□ Rice<br>□ Quinoa<br>□ Amaranth<br>□ Crunchy / muesli<br>□ Other:<br>…………………………………….	□ Wheat<br>□ Wholemeal rye<br>□ Light rye<br>□ Buckwheat<br>□ Corn<br>□ Own bake<br>□ Other:<br>…………………………………….	□ Natural yogurt<br>□ Cottage cheese<br>□ Cheese<br>□ Mozarella<br>□ Feta cheese<br>□ Cream cheese<br>□ Parmezan<br>□ Camembert<br>□ Blue cheese<br>□ Greek yogurt<br>□ Kefir<br>□ Buttermilk<br>□ Other:<br>……………………………………..
**Dairy goat/sheep + % fat**	**Plant products + % fat**	**Meat products**
□ Goat/Sheep yogurt<br>□ Goat/Sheep cheese<br>□ Goat/Sheep feta cheese<br>□ Cream cheese<br>□ Pecorino Romano cheese<br>□ Other:<br>…………………………………	□ Soy drink<br>□ Soy yogurt<br>□ Coconut yogurt<br>□ Cream: soy, oat, rice, almond<br>□ Drink: oat, rice, coconut-rice, almond, millet<br>□ Other:<br>……………………………………..	□ Chicken: breast, loin, legs<br>□ Turkey: breast, loin, legs<br>□ Pork loin<br>□ Ground pork<br>□ Beef<br>□ Lamb<br>□ Veal<br>□ Duck/ goose<br>□ Wild meat<br>□ Chicken/pork liver

		□ Other: ……………………………………..
**Other meat products**<br><br>□ Cold meat: from poultry, pork<br>□ Kabanos: poultry, pork<br>□ Sausages<br>□ Liverwurst<br>□ Other: ……………………………………..	**Fish**<br><br>□ Salmon<br>□ Trout<br>□ Cod, sole fish, minty zander, bream<br>□ Flounder<br>□ Tuna (steak)<br>□ Tuna in water/oil<br>□ Sprat, sardine<br>□ Herring<br>□ Smoked fish: salmon, Trout, Cod, Tuna<br>□ Prawn<br>□ Sushi<br>□ Other seafood<br>□ Other: ………………………………………..	**Eggs**<br><br>□ Chicken<br>□ Hard boiled<br>□ Soft-boiled<br>□ Scrambled<br>□ Omlete<br>□ Fried<br>□ Quail<br>□ Other: ……………………………………
**Carbohydrates**<br><br>□ Potatos<br>□ Batats<br>□ Brown rice<br>□ White rice<br>□ Wholemeat pasta<br>□ Wheat pasta<br>□ Egg-free pasta<br>□ Rice pasta<br>□ Soy pasta<br>□ Mung pasta<br>□ Other types of pasta<br>□ Other: ………………………………	**Other carbohydrates**<br><br>□ Buckwheat groats<br>□ Millow groats<br>□ Wheat groats<br>□ Spelled groats<br>□ Barley<br>□ Couscous from durum wheat/ corn<br>□ Quinoa<br>□ Amaranth<br>□ Wheat pancakes<br>□ Wholemeat pancakes<br>□ Flour pancakes<br>□ Tortilla<br>□ Other: ………………………………………	**Vegetarian products**<br><br>□ Tofu<br>□ Soy cutlets<br>□ Tempeh<br>□ Vegetables sandwich creams<br>□ Hummus<br>□ Natto<br>□ Other: …………………………………….
**Vegetables**<br><br>□ Tomato<br>□ Cucumber<br>□ Pepper<br>□ Radish, kohirabi, zucchini, pumpkin, eggplant<br>□ Broccoli, cauliflower<br>□ Beetroot<br>□ Corn<br>□ Cabbage<br>□ Brussels, sprouts<br>□ Lettuce, spinach, rucola, chicory<br>□ Carrot<br>□ Parsley<br>□ Celery<br>□ Onion, garlic<br>□ Dill, chives<br>□ Sprouts<br>□ Coriander, basil<br>□ Ginger<br>□ Pattison<br>□ Kale	**Pulses**<br><br>□ Green bean<br>□ Peas<br>□ Green peas<br>□ White/red bean<br>□ Lentils<br>□ Broad bean<br>□ Soy<br>□ Other …………………………………….	**Dried tropical food and other**<br><br>□ Sunflower seeds<br>□ Pumpkin seeds<br>□ Sesame<br>□ Coconut shreds<br>□ Almonds<br>□ Walnuts<br>□ Cashews<br>□ Pistaccio<br>□ Hazelnuts<br>□ Peanuts<br>□ Raisins<br>□ Figs<br>□ Cranberry<br>□ Apricot<br>□ Chia seeds, linseed<br>□ Wheat bran<br>□ Other: ……………………………………..

□ Other: ……………………………………..		
**Soups**<br><br>□ Cucumber<br>□ Tomato<br>□ Vegetable<br>□ Chicken soup<br>□ Borsch<br>□ Bean soup<br>□ Barley<br>□ Mushroom soup<br>□ Cream soups<br>□ Instant soups<br>□ Other<br>…………………………………….	**Adds**<br><br>□ Olives: green and black<br>□ Dried tomatos<br>□ Capers<br>□ Tomato souce, passata<br>□ Ajvar<br>□ Balsamic vinegar<br>□ Soy souce<br>□ French dressing<br>□ Yogurt sauce<br>□ Mustard<br>□ Ketchup<br>□ Horseradish<br>□ Honey<br>□ Mayonaisee<br>□ Other:<br>………………………………………	**Herbs**<br><br>□ Curry<br>□ Sweet/ Spicy pepper<br>□ Ginger<br>□ Provence herbs<br>□ Basil<br>□ Oregano<br>□ Thyme<br>□ Chilli<br>□ Turmeric<br>□ Curry paste<br>□ Rosemary<br>□ Cinamon<br>□ Vanilia<br>□ Pepper<br>□ Other:<br>……………………………………..
**Fruits**<br><br>□ Apple, pear<br>□ Orange<br>□ Grapefruit<br>□ Ananas, mango, kaki, papaya<br>□ Grapes<br>□ Avokado<br>□ Rhubarb<br>□ Redcurrant, white currant<br>□ Pomegranate<br>□ Mango<br>□ Banana<br>□ Plum<br>□ Peach, nectarine<br>□ Watermelon, melon<br>□ Kiwi<br>□ Strawberry, raspberry, berries, blueberries<br>□ Cherries<br>□ Other:<br>…………………………………….	**Fats**<br><br>□ Soy oil<br>□ Rapeseed oil<br>□ Cottonseed oil<br>□ Olive oil<br>□ Margarine<br>□ Butter<br>□ Clarified butter<br>□ Masmix<br>□ Grape seed oil<br>□ Linen oil<br>□ Coconut oil<br>□ Other:<br>……………………………………….	**Other:**<br><br>□ Fruit/vegetables coctails<br>□ Juice<br>□ Tea<br>□ Mushrooms<br>□ Fruit in sirup<br>□ Marmalades<br><br>……………………………………

Signature of the patient confirms that all the data filled in the questionnaire are consistent with the truth and his/her state of knowledge on the day of fulfilling the questionnaire.
I consent to the processing of my personal data for research purposes according to the regulations of the Bill of August 29, 1997, concerning personal data protection *(Dz. U. z 2002 r. nr 101, poz. 926, with alterations).*

Date of investigation: ……………… *Patient's signature:………………………………………*
